# Impact of molecular subtypes on the prediction of distant recurrence in estrogen receptor (ER) positive, human epidermal growth factor receptor 2 (HER2) negative breast cancer upon five years of endocrine therapy

**DOI:** 10.1186/s12885-019-5890-z

**Published:** 2019-07-15

**Authors:** Mark Laible, Kerstin Hartmann, Claudia Gürtler, Tobias Anzeneder, Ralph Wirtz, Stephan Weber, Thomas Keller, Ugur Sahin, Martin Rees, Annette Ramaswamy

**Affiliations:** 1 BioNTech Diagnostics GmbH, An der Goldgrube 12, 55131 Mainz, Germany; 2 PATH Biobank, Schäftlarnstr. 62, 81337 Munich, Germany; 3 Stratifyer Molecular Pathology GmbH, Werthmannstr. 1c, 50935 Köln, Germany; 4 ACOMED Statistik, Fockestraße 57, 04275 Leipzig, Germany; 5 Gemeinschaftspraxis für Pathologie, Brustzentrum am St.-Johannes-Hospital, Amalienstraße 21, 44137 Dortmund, Germany; 6 Institut für Pathologie, Universitätsklinikum Giessen und Marburg, Baldingerstraße, 35043 Marburg, Germany

**Keywords:** Breast cancer, Luminal A-like, ER, *MKI67*, Ki67, Distant recurrence

## Abstract

**Background:**

Current evidence suggests that patients with Luminal A early breast cancer can skip chemotherapy or extended endocrine therapy, but immunohistochemistry-based biomarker analysis for St Gallen subtyping may not be reproducible. We asked whether RT-qPCR can be used instead to address this clinical question.

**Methods:**

RNA was extracted from tumor material derived from ER+/HER2- patients receiving adjuvant endocrine treatment for low-risk cancers and was semi-quantified by RT-qPCR with the MammaTyper®. St Gallen subtypes were based on the mRNA expression of *ERBB2*/HER2*, ESR1*/ER*, PGR*/PR and *MKI67*/Ki67 after dichotomizing at predefined cut-offs. Differences in distant disease-free survival (DDFS) were assessed by Kaplan Meier analysis and Cox regression.

**Results:**

With a median follow up of 7.8 years, there were ten events in the group of 195 *Luminal A-like* tumors (5.1%) and 18 events in the remaining 127 tumors (14.1%), consisting mostly of *Luminal B-like* cases (*N* = 119). *Luminal A-like* had significantly better DDFS over the entire follow-up period (HR 0.35, 95% CIs 0.16–0.76, *p* = 0.0078) with a trend towards reduced probability of recurrences also in the late phase (> 5 years) (HR 0.20, *p* = 0.052). The survival advantage spanning the entire follow-up period persisted in the pN0 or pN0-N1 subgroups or after correcting for clinicopathological parameters. *MKI67* alone significantly predicted for worse DDFS (HR 2.62, 95% CIs 1.24–5.56, *p* = 0.0088).

**Conclusions:**

St Gallen *Luminal A-like* tumors identified by RT-qPCR display markedly low rates of distant recurrence at ten years follow-up. Patients with such tumors could be spared chemotherapy due to the obviously unfavourable benefit/toxicity ratio.

## Background

The survival of patients who undergo surgery for early-stage estrogen receptor (ER)-positive/human epidermal growth factor receptor 2 (HER2)-negative breast cancers is heterogeneous. Whereas five years of endocrine treatment suffices for many women, others remain at significant risk of early or late distant recurrence warranting additional chemotherapy or extended endocrine therapy [1–3]. Accurate differentiation between these clinical subsets of early breast cancer impacts the health and quality of life of thousands of women worldwide and remains one of the most intensively investigated areas in the field of breast cancer biomarkers. Driving this campaign is the fact that calculation of recurrence risk by traditional clinicopathological prognostic factors such as histologic grade and Ki67 lacks necessary accuracy and reproducibility [4].

The discovery of a natural transcriptional architecture of breast cancer in the form of the “intrinsic” subtypes had a tremendous impact on the prognostic stratification of ER-positive/HER2-negative disease, showing that an accurate distinction between a low-risk Luminal A and a high-risk Luminal B group of tumors is possible by means of measuring gene expression [5, 6]. During the last two decades many gene expression assays have gradually entered the clinic as there is compelling evidence that their routine use refines residual risk estimates obtained by traditional clinicopathological risk factors alone [7].

Owing to increased costs of commercial multi-gene assays, global initiatives have proposed immunohistochemistry (IHC) as a means for approximating the biological signals stored within mRNA expression [8]. According to the updated 2013 St Gallen classification of breast cancer, “intrinsic” subtypes may be sufficiently profiled by ER, progesterone receptor (PR), HER2 and marker of proliferation Ki67 protein expression [9]. In this evolving concept, tumors whose growth depends on ER but not HER2 may be either *Luminal A-like* or *Luminal B-like*, with the two subtypes differing in their expression of PR and Ki67. The distinction can be used to guide treatment decisions in the adjuvant setting, with higher proliferation and lower ER activity (characteristic of *Luminal B-like* tumors) indicating decreased 5- or 10-year (distant)-recurrence-free probabilities and increased benefit from chemotherapy and possibly extended endocrine therapy [2, 3].

Although more economical, the St Gallen subtypes are highly sensitive to the quality of local testing and scoring IHC practices. Efforts to raise quality standards are still ongoing particularly for Ki67, owing to the essential link between tumor proliferation and chemotherapy response, which however may be impossible to harness in practice due to inherent difficulties with Ki67 scoring. [10, 11]. Overall, reproducibility studies have not yet provided clear guidance on the validity of Ki67 immunostaining and data drawn from actual routine samples is missing [12]. These shortcomings continue to stimulate the search for new prognostic biomarkers with enhanced analytical performance characteristics and economical profile.

In previous studies we have shown that St Gallen molecular subtyping is also informative when using reverse transcription quantitative real-time polymerase chain reaction (RT-qPCR) for measuring the mRNA expression of the 4 biomarkers, i.e. ER (*ESR1*), PR (*PGR*), HER2 (*ERBB2*) and Ki67 (*MKI67*) [13, 14]. Using predefined cut-off values for separating high from low (positive from negative) gene expression, the resulting subtypes are stable against various analytical perturbations across different laboratories and operators [15, 16]. Our approach leverages the analytical advantages of RT-qPCR, to enable guideline-driven, standardized and interpretation-free breast cancer molecular classification. Herein we asked whether the subtypes obtained by MammaTyper® can stratify patients in prognostic subgroups in a cohort of clinical low-risk ER-positive/HER2-negative breast cancers treated with standard 5-year endocrine therapy.

## Methods

This is a retrospective observational study on formalin-fixed paraffin-embedded tumor material provided by the non-profit organization PATH Biobank (http://www.path-biobank.org/index.php/en/about-path/) [17]. Inclusion criteria were the following: women diagnosed consecutively between 2005 and 2011 and treated with surgery and hormonal systemic therapy (no chemotherapy) in all seven certified breast cancer centers cooperating with PATH Biobank in Germany, adequate tumor material obtained during routine surgery and ER-positive/HER2-negative breast cancer assessed by local protocols.

The informed consent documents, especially regarding sample donation and data processing, were reviewed by the Bavarian State Office for Data Protection Supervision Authority and approved by the ethics committee of the Medical Faculty of the Rheinischen Friedrich-Wilhelms-Universität Bonn, Germany (vote number: 255/06). All participants gave written informed consent as per PATH Biobank policy.

Tumor cell content (TCC) was determined on a 3 μm H&E stained slide as the planimetric ratio of areas covered by invasive carcinoma in relation to areas covered by DCIS and non-neoplastic tissue. For samples with at least 20% TCC, RNA was extracted from a single 10 μm section using the bead-based RNA purification kit (RNXtract®, BioNTech Diagnostics GmbH) according to the manufacturers’ instructions. For RT-qPCR, the MammaTyper® in vitro diagnostic assay (BioNTech Diagnostics GmbH) was used according to manufacturers’ instructions on a CFX96™ system (BioRad) using total RNA from RNXtract® eluates. A robust detection of the two reference genes *CALM2* and *B2M* per pre-defined sample validity criteria was used as a quality control for the RNA samples. Gene expression levels for *ERBB2*, *ESR1*, *PGR* and *MKI67* were categorized as being either positive or negative based on predefined cut-off values [18]. Thereafter, each case was classified according the St Gallen surrogate definitions for molecular subtypes (Table 1).

**Table 1:** Translation of MammaTyper® single marker results into molecular subtypes according to St Gallen classification (2013)

*ERBB2*	*ESR1*	*PGR*	*MKI67*	St Gallen Subtype
pos	pos	pos	pos	*Luminal B-like (HER2 positive)*
pos	pos	pos	neg	*Luminal B-like (HER2 positive)*
pos	pos	neg	pos	*Luminal B-like (HER2 positive)*
pos	pos	neg	neg	*Luminal B-like (HER2 positive)*
pos	neg	pos	pos	*Not defined by St Gallen (ER−/PR+)*
pos	neg	pos	neg	*Not defined by St Gallen (ER−/PR+)*
pos	neg	neg	pos	*HER2 positive (non-luminal)*
pos	neg	neg	neg	*HER2 positive (non-luminal)*
neg	pos	pos	pos	*Luminal B-like (HER2 negative)*
neg	pos	pos	neg	*Luminal A-like*
neg	pos	neg	pos	*Luminal B-like (HER2 negative)*
neg	pos	neg	neg	*Luminal B-like (HER2 negative)*
neg	neg	pos	pos	*Not defined by St Gallen (ER−/PR+)*
neg	neg	pos	neg	*Not defined by St Gallen (ER−/PR+)*
neg	neg	neg	pos	*Triple negative (ductal)*
neg	neg	neg	neg	*Triple negative (ductal)*

The main measure of outcome was distant disease-free survival (DDFS), defined as any recurrence at a distant organ. Deaths without prior documentation of recurrence were scored as censored events. The analysis was carried out in the entire population and separately in patients with node-negative breast cancer (pN0), or patients with 0 to 3 positive lymph nodes (pN0 and pN1). In all analyses we considered patients whose tumors were classified as *Luminal A-like* as belonging to the low risk group, whereas the remaining subtypes were high risk. The principal objective was to compare the two prognostic subsets for the entire (0–10 years) and for the late (5–10 years) follow-up periods. In addition, we performed Cox regression to assess the value of the RNA-based St Gallen risk stratification compared to tumor size, nodal status and histologic grade. For this analysis, covariables were treated as continuously scaled parameters. Finally, we tested whether *MKI67* as a single marker would suffice for prognostication.

Double pseudonymized clinicopathological data were sent directly from PATH Biobank to ACOMED Statistik (Leipzig, Germany) and were inaccessible by individuals generating MammaTyper® results. All analyses were carried out in SAS 9.4. *P*-values < 0.05 were considered significant.

## Results

**Fig. 1:**
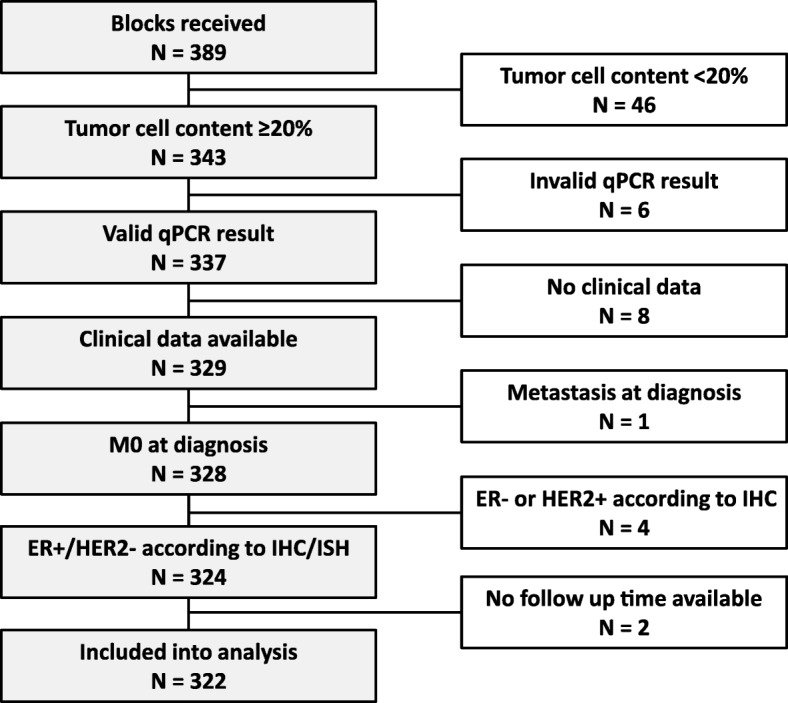
Flow of sample selection and dropout: Most invalidated samples were due to insufficient tumor cell content. A minimum content of 20% was applied in line with MammaTyper® published specifications [15]

Cohort assembly and sample dropout are presented in Fig. 1. Median follow up was 7.8 years, during which 28 distant events occurred (8.5%). Standard clinicopathological characteristics are listed in Table 2. This is a low-risk cohort of mainly postmenopausal women with G1–2, pT1–2, pN0–1 invasive breast cancer consisting of primarily *Luminal A-like* (60.6%) and *Luminal B-like* cancers (37%). As expected, some discordance was found between original IHC classification and PCR, with 2 cases being reclassified as *Luminal B-like (HER2 positive*) and 4 as *Triple negative*. Women with *Luminal A-like* breast cancers were at low risk of developing a distant recurrence after ten years of follow-up (5.1%), whereas in the group of *Luminal B-like*, 13.4% of cancers recurred with distant metastases (Table 3). The negative predictive value of the stratification was 94.9% (only ten patients out of 195 being classified as *Luminal A-like* would be expected to develop metastases within ten years).

**Table 2:** Patient demographics and sample characteristics

Age [years]		
Min	37.7	
P25%	55.7	
Median	62.46	
P75%	69.3	
Max	83.5	
	N	%
Menopausal status		
Premenopausal	32	9.9
Perimenopausal	6	1.9
Postmenopausal	258	80.1
NA	26	8.1
Breast-conserving surgery		
Yes	279	86.6
No	43	13.4
Histological subtype		
Invasive ductal	243	75.5
Invasive lobular	63	19.6
Tubular	7	2.2
Mucinous	5	1.6
Others	4	1.2
Tumor grading		
G1	83	25.8
G2	224	69.6
G3	15	4.7
T-stage		
pT 1a	5	1.6
pT 1b	72	22.4
pT 1c	185	57.5
pT 2	56	17.4
pT 3	4	1.2
N-stage		
pN 0	278	86.3
pN 1mic	13	4
pN 1a	25	7.8
pN 2a	2	0.6
pN 3a	3	0.9
NA	1	0.3

**Table 3:** Distribution of MammaTyper® subtypes according to distant events

			no distant event		with distant event	
	N	%	N	%	N	%
*Luminal A-like*	195	60.6	185	94.9	10	5.1
*Luminal B-like (HER2 negative)*	119	37.0	103	86.6	16	13.4
*Luminal B-like (HER2 positive)*	2	0.6	2	100	0	0
*Triple negative (ductal)*	4	1.2	3	75.0	1	25.0
Not defined by St Gallen (*ESR1*−/*PGR*+)	2	0.6	1	50.0	1	50.0
Total	322	100	294	91.3	28	8.7

**Fig. 2:**
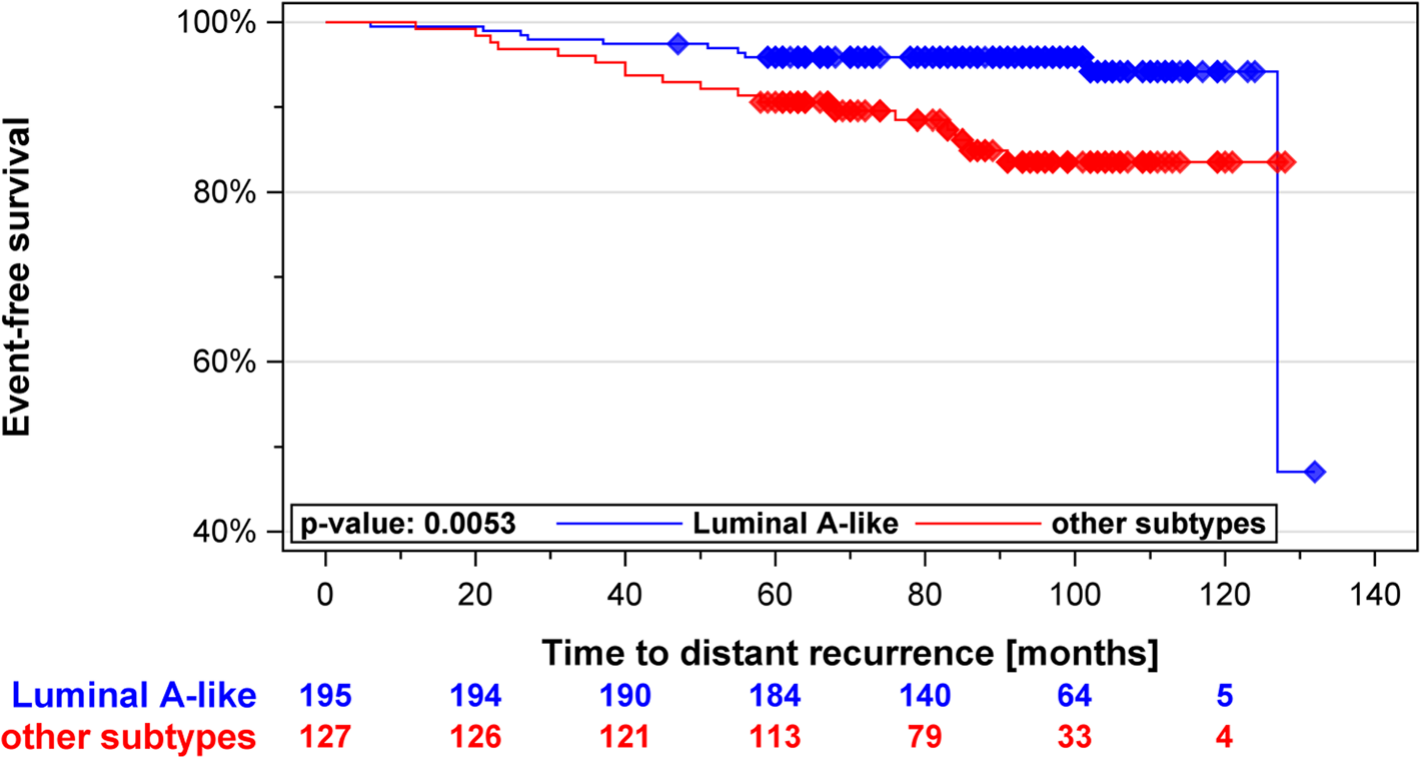
Kaplan Meier curves of MammaTyper® *Luminal A-like* vs. other subtypes as one group: *Luminal A-like* cases were statistically significantly associated with better distant disease-free survival

In Kaplan-Meier analysis we observed a significant difference in ten-year DDFS between *Luminal A-like* and other subtypes grouped together as one (Fig. 2). *Luminal A-like* tumors were associated with 65% reduction of risk of distant recurrence compared to the non-*Luminal A-like* group (HR 0.35; CI 0.16–0.76, p = 0.0078). Estimates of HR were similarly significant when focusing on patients with 0–3 lymph nodes, when restricting the analysis to node-negative breast cancer patients or when the comparison was between *Luminal A-like* and *Luminal B-like* tumors (Table 4). In multivariable analysis, the prognostic information provided by the subtypes remained significant after adjusting for nodal status, tumor size and tumor grade (Table 5). T-stage was the only conventional parameter that remained significant in the final model.

**Table 4:** Cox regression and log rank analysis: *Luminal A-like* vs. other subtypes

		Hazard Ratio			Log-Rank Test
Effect	Subset	Estimate	95% CIs	*p*-value	*p*-value
*Luminal A-like* vs. other subtypes	all patients	0.350	0.16–0.76	0.0078	0.0053
	0–3 positive nodes	0.370	0.16–0.85	0.0184	0.0141
	node negative	0.344	0.14–0.86	0.0234	0.0177
*Luminal-A-like* vs. *Luminal B-like*	all patients	0.37	0.19–0.81	0.0141	0.0106

**Table 5:** Multivariable Cox regression analysis for Tumor subtype *Luminal A-like*

		Hazard Ratio		
Parameter	Effect	Estimate	95% CIs	*p*-value
Tumor subtype *Luminal A-like*	*Luminal-A-like*	0.415	0.180–0.959	0.0395
T-stage	Increase by ‘+ 1’	2.137	1.201–3.802	0.0098
N-stage	Increase by ‘+ 1’	1.540	0.933–2.539	0.0910
Tumor grade	Increase by ‘+ 1’	1.897	0.778–4.627	0.1591

The *Luminal A-like* subtype was a favourable prognostic indicator associated with a reduced probability of recurrences also in the late (> 5 years) follow-up period with an HR of 0.2. However, as shown by the wider CIs (0.041–1.015) and by the marginally significant *p*-value (0.052), this association lacked the strength observed over the entire follow-up period.

**Fig. 3:**
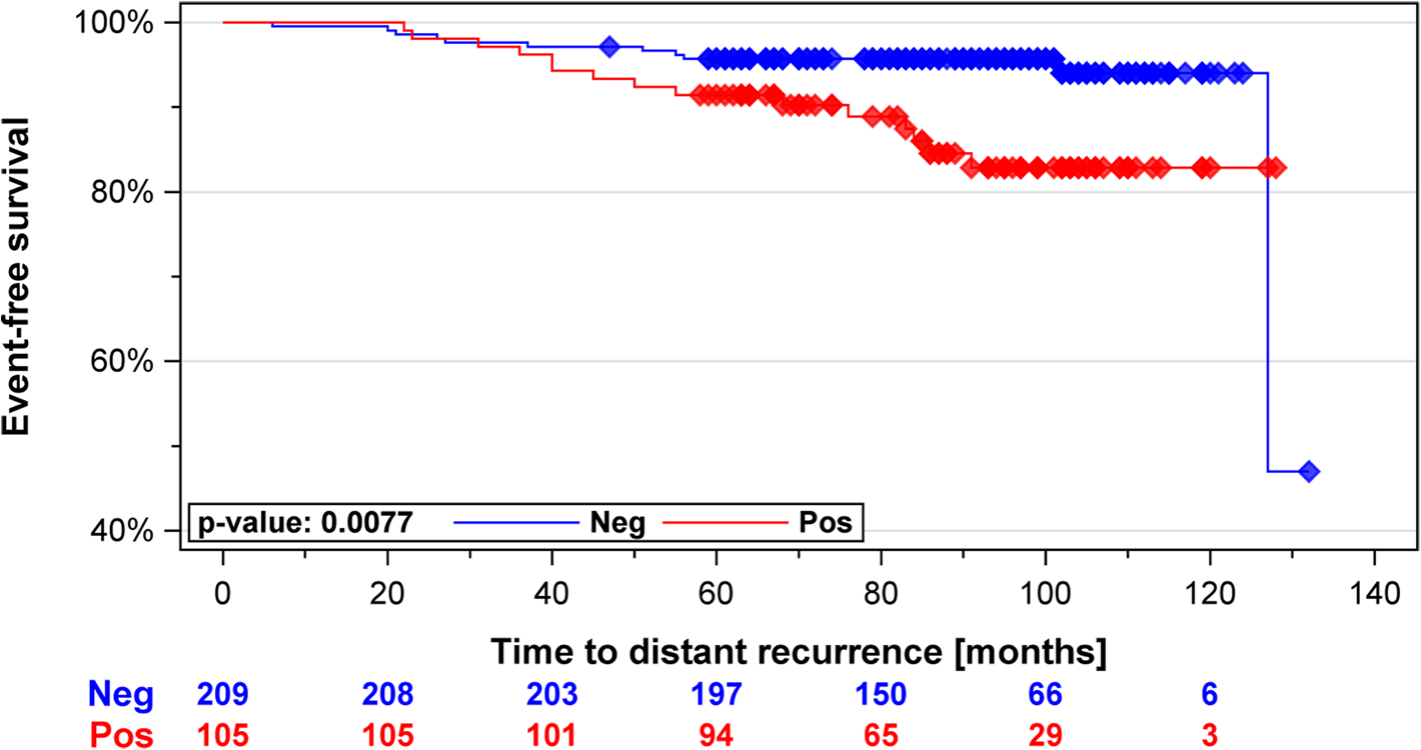
Kaplan Meier curves of MammaTyper® *MKI67* positive (high) vs. *MKI67* negative (low) in *ESR1+/ERBB2-* tumors: *MKI67* negative cases were statistically significantly associated with better distant disease-free survival

According to the St Gallen surrogate definitions of the molecular subtypes, *Luminal B-like* cancers consist of ER-positive tumors that either display high Ki67 irrespective of PR or alternatively lack both Ki67 and PR expression, illustrating the increased weight of tumor proliferation in this classification. For this reason, we further investigated whether *MKI67* as a single marker could be used for stratifying patients into prognostic groups. High *MKI67* expression was associated with a higher risk of distant recurrence over all patients (*N* = 322, HR 2.62, 95% CIs 1.24–5.56, *p* = 0.0088) as well as in *ESR1+/ERBB2-* patients (*N* = 314, HR 2.77, 95% CIs 1.27–6.03, *p* = 0.0077) (Fig. 3). In multivariable analysis in the *ESR1+/ERBB2-* set the *MKI67* status and tumor stage displayed almost identical hazard ratios, however only stage was clearly significant (Table 6).

**Table 6:** Multivariable Cox regression analysis for *MKI67* low/high

		Hazard Ratio		
Parameter	Effect	Estimate	95% CIs	*p*-value
*MKI67* Positive (high)	Positive	2.270	0.986–5.226	0.0541
T-stage	Increase by ‘+ 1’	2.273	1.274–4.052	0.0054
N-stage	Increase by ‘+ 1’	1.401	0.819–2.399	0.2187
Tumor grading	Increase by ‘+ 1’	2.111	0.814–5.478	0.1245

## Discussion

Estimating the prognosis of patients with early-stage ER-positive/HER2-negative breast cancer treated with standard 5-year endocrine therapy is essential for assessing the need for additional adjuvant systemic treatment. Herein we asked whether St Gallen definitions of molecular subtypes can be combined with RT-qPCR to successfully stratify patients in prognostic groups based on the expression of *ERBB2*, *ESR1*, *PGR* and *MKI67* in their tumors*.* We show that the outcome of *Luminal A-like* tumors significantly differed from the rest of the cohort independent of conventional clinicopathological parameters and to such extent as to render benefit from adjuvant chemotherapy unlikely, especially when counting potential toxicities. These results reiterate the importance of St Gallen definitions of “intrinsic” subtypes, particularly the distinction between *Luminal A-like* and *Luminal B-like* cancers, for predicting the outcome of ER-positive disease and for assessing the need for adjuvant chemotherapy [3]. Interestingly, subtyping by RT-qPCR remained significant after correcting for clinicopathological variables, whereas *MKI67* RNA expression alone did not, which is consistent with subtypes being more informative as they integrate several biological signals quantified by the individual markers.

The favourable prognosis of Luminal A breast cancers has been invariably demonstrated in diverse patient populations and treatment settings, including ER-positive low-risk cohorts [19–23]. Luminal A tumors are characterized by low proliferation and high expression of ER-regulated genes and are thus resistant to cytotoxic therapy, as also illustrated by the lack of pathological complete response after neoadjuvant chemotherapy despite excellent 5-year survival rates [24]. Consequently, accurate and precise classification of the Luminal A subtype allows patients with such tumors to safely forego chemotherapy and be spared associated toxicities [2].

Stratifying tumors into Luminal A or Luminal B breast cancer, may also help predict the risk of distant recurrence at years 5–10 of follow-up by being prognostic in this timespan [25] and may thus serve as a biomarker for or against endocrine treatment continuation [26]. In the present study, *Luminal A-like* tumors demonstrated a trend towards improved outcomes in the late follow-up period; however, results did not reach statistical significance. Prediction of recurrence risk during late follow-up remains an area of active investigation with evidence suggesting that some molecular profiling tests may per-design perform differently in this timespan [7]. More data will be needed to answer this question for the herein proposed RNA-based subtyping, as sample and event numbers in the corresponding subgroup analysis of years 5–10 was not enough for drawing a definite conclusion.

The ability of genomic assays for prognostication carried out under high quality standards and without bias from human interpretation often comes with a price tag, which in several parts of the world may be too high for both patients and health systems [8]. Affordable alternatives exploiting conventional clinicopathological parameters to approximate the output of gene-expression tests are therefore becoming popular [9, 27, 28]. In clinical reality however, parameters such as histologic grade and IHC for Ki67 or ER, regularly required as input, are still infested by significant levels of inter-observer variability [29–35]. Such shortcomings inevitably weaken the real-life utility of pathology-based proxies of molecular profiling, a challenge that could be overcome using biomarkers with an improved analytical performance record such as RT-qPCR.

Previously we have shown that St Gallen subtyping carried out with RNA- instead of protein expression significantly interacts with the benefit of docetaxel in the adjuvant setting and that *MKI67* expression may enhance the prediction of pathological complete response [13, 14]. In the present work our approach successfully identified patients with luminal tumors whose risk of relapse at ten years is so low that benefit from chemotherapy would be unable to surpass the potential adverse effects. Alongside an expanding list of evidence on clinical validity, gene expression of *ERBB2*, *ESR1*, *PGR*, and *MKI67* with RT-qPCR has a noticeable track record of both analytical precision and reproducibility in the context of decentralized biomarker testing [15, 16]. These features may advocate an enhanced role of RT-qPCR in breast cancer biomarker testing.

Despite being subject to selection bias due to its retrospective nature, our study bears similarity to the TRANSATAC and ABCSG-6 and -8 trial populations that were used to prospectively validate many prognostic gene signatures [36, 37]. Moreover, none of the patients received either chemotherapy or targeted therapy, while specimen archiving and data collection was carried out according to standard operating procedures. Nevertheless, a prospective study remains the golden standard for demonstrating the clinical validity of a biomarker and thus prospective validation should be the long-term goal for MammaTyper®. Additional strengths of our study are the use of predefined analysis methods, including cut-off values and the fact that subtypes were generated by investigators blinded to clinical variables or follow-up. A limitation of our work is that multivariable analysis did not include IHC data for Ki67, as these were not available by PATH Biobank due to the fact that Ki67 has not been a standard parameter in routine pathological testing throughout the diagnosis years of the patients included in this study. Lastly, due to the small number of patients with 1–3 lymph nodes, we were not able to test our hypothesis separately in this clinical subset. Even though the favourable effect of the *Luminal A-like* subtype remained constant between node-negative patients and the entire cohort, at this point it is not possible to conclude whether patients with 1–3 lymph nodes could be spared chemotherapy. Several gene expression prognostic tests such as MammaPrint®, Oncotype DX® and Prosigna® have been studied extensively for years. The evidence provided by these studies shows that the prognostic information from different tests is broadly equivalent for the population of women with estrogen receptor -positive breast cancers. On the individual patient level however, the established tests may provide differing risk categorization and subtype information [38]. This observed disagreement raises the question how the test results impact treatment decisions for individual patients [38]. These concerns are one of the reasons why the patient and health economic benefits of the individual prognostic tests is still under intense discussion. The question of individual patient management therefore needs to be addressed in future research together with the question whether a low cost molecular assay measuring fewer parameters may provide similar prognostic information while being accessible to a broader range of patients.

## Conclusions

Using RNA instead of protein profiles to classify tumors according to the St Gallen surrogate definitions of molecular subtypes provides independent prognostic information in early-stage ER-positive/HER2-negative breast cancer. *Luminal A-like* tumors displayed markedly low rate of distant recurrence at ten years of follow-up, indicating lack of benefit from the addition of chemotherapy. Given the importance of *MKI67* in separating *Luminal A-like* from *Luminal B-like* tumors, our study lends further support to the clinical significance of tumor proliferation and particularly *MKI67* gene expression for the management of breast cancer.

## Data Availability

The datasets used and/or analyzed during the current study are available from the corresponding author on reasonable request.

## Abbreviations

DDFS: Distant disease-free survival
ER: Estrogen receptor
ERBB2: Epidermal growth factor receptor 2
ESR1: Estrogen receptor 1
FFPE: Formalin-fixed paraffin-embedded
H&E: Hematoxylin and eosin
HER2: Epidermal growth factor receptor 2
IHC: Immunhistochemistry
MKI67: Marker of proliferation Ki-67
mRNA: messenger RNA
PATH: Patients’ Tumor Bank of Hope
PGR: Progesterone receptor
PR: Progesterone receptor
RNA: Ribonucleic acid
RT-qPCR: Reverse transcription quantitative real-time PCR
TCC: Tumor cell content
